# Agreement of young adults and orthodontists on dental aesthetics & influencing factors of self-perceived aesthetics

**DOI:** 10.1186/s12903-018-0575-6

**Published:** 2018-06-19

**Authors:** Ying Cai, Wulong Du, Feiou Lin, Shengjia Ye, Yanling Ye

**Affiliations:** 1 Department of Orthodontics, School and Hospital of Stomatology, Wenzhou Medical University, West Xueyuan Road 373, Wenzhou, Zhejiang, 325000 People’s Republic of China; 2 School of Stomatology, Wenzhou Medical University, Wenzhou, Zhejiang, People’s Republic of China; 3 School and Hospital of Stomatology, Shandong University, Jinan, Shandong People’s Republic of China

**Keywords:** IOTN, Aesthetic component, Personality, Agreement

## Abstract

**Background:**

The aim of this study is to assess the agreement between orthodontist’s and Chinese young adult’s self-perceived aesthetics, the normative treatment need based on the Index of Orthodontic Treatment Need (IOTN), and the main factors affecting the self-perceived aesthetics.

**Methods:**

A random sample of 348 Chinese young adults (116 males and 232 females) aged 17–24 years were recruited in this study. Two orthodontists were involved in rating the cases. Orthodontic treatment need was assessed according to the Index of Orthodontic Treatment Need, including Aesthetic components and Dental Health Components (AC and DHC). Personality traits were assessed according to Eysenck Personality Questionnaire (EPQ). Cohen’s kappa test was used to assess the agreement, and spearman’s correlation coefficient was used to analyze the association among all variables.

**Results:**

A statistically significant level of agreement was observed between young adult’s perception and orthodontist’s perception in IOTN (kappa = 0.14). A positive relationship (*p* < 0.001)existed between the young adult’s AC and the orthodontist’ s AC (*r* = 0.275), and between the young adult’ s AC and the normative need DHC (*r* = 0.195). The orthodontist’s AC was strongly related to the normative need (*r* = 0.743, *p* < 0.001). Association between the young adult’s AC and gender and EPQ-E were also observed.

**Conclusions:**

Young adults tend to be less critical in assessing orthodontic treatment needs than orthodontists. The orthodontist’s AC reflecting subjective treatment need is strongly connected to the normative need. The adult’s perception of aesthetic component is affected by factors such as gender and personality traits.

## Background

The subjective orthodontic treatment need of Chinese natives is high [1]. Apart from the dental health needs, it is necessary to take subjective aesthetic into consideration as well [2]. Among the Chinese young adults, the common motivation for orthodontic treatment is associated with the desire for improvement in appearance [3]. An attractive facial appearance has a positive impact on socio-psychological well-being as well as professional relations [4]. It is also suggested that dental esthetics may be important in individual oral-health attitudes and behaviors [5–7]. When young adults are seeking orthodontic treatment, it is appropriate for orthodontists to consider the patient’s aesthetic component, since this component may influence the communication between the patient and the orthodontist, which will in turn enable the orthodontist to assess his or her expectations. The self-perceptions of young adults are important indicators when deciding to undertake treatment, and this might complement conventional clinical evaluation. However in practice, the patient’s own perception is often ignored by most orthodontic treatment need indices [8].

The perceptions of esthetics maybe differ between laypersons and orthodontists. Many studies reveal that the patient’s perception is not aligned with the orthodontist’s opinion [9–12]. They thought that the professional opinions of orthodontists are generally more critical than those of laypersons when assessing dento-facial esthetics. However, few authors have investigated the self-perceptions of dental esthetics among young adults.

In recently, many orthodontic need questionnaires have been developed and used as outcome measures. The index of orthodontic treatment need (IOTN) consists of the Aesthetic Component (AC) and the Dental Health Component (DHC), being used to rank malocclusion. AC is the subjective part, associated with the assessment of malocclusion [13]. Although, the previous study showed there is only a moderate agreement between AC and DHC [14].This difference between the DHC and AC reflects that AC assesses the aesthetic aspects of the malocclusion, only in frontal view, and highlights the subjective nature of it. Therefore, any clinician who is interested in using the IOTN should receive proper training and undergoes the calibration process [15]. However, most researchers found that the IOTN was considered a reproducible and effective way to ascertain the patient’s perception of the esthetics [16–20].

Thus, the aim of this study is to assess the agreement between orthodontist’s and Chinese young adult’s self-perceived aesthetics, the normative treatment need based on the Index of Orthodontic Treatment Need (IOTN), and the main factors affecting the self-perceived aesthetics.

## Methods

All of the protocols were reviewed and approved by the Health Research Ethics Board of Wenzhou Medical University. Written informed consent (including the release for dental records) was obtained from each participant.

### Subjects

It was estimated that a sample size of 48 subjects would be needed to demonstrate a significant change in OHRQoL, with an 80% probability power at the 5% level of significance. The sample size was inflated by a 10% margin to allow for loss to follow-up and dropouts; thus, the total sample size was a minimum of 52.

A total of 348 young adults (116 males and 232 females) aged 17–24 years (mean 20.37 ± 1.22 years) were randomly selected from two universities in Wenzhou, China from March 2017–May 2017. The distribution of gender was balanced: males represented 33.3% and females 66.7% of the sample population (Table 1). Exclusion criteria included patients with cognitive disorders or chronic medical conditions, those who had previously received any type of orthodontic treatment, and those with craniofacial anomalies such as cleft lip and palate. Participants who did not consent or were undergoing orthodontic treatment were also not included in the study. The data was collected from a clinical inspection by the two orthodontists. All of the questionnaires were filled out within a 30-min time frame and collected on the spot. The questionnaires included the index of orthodontic treatment need (IOTN), Eysenck Personality Questionnaire (EPQ).

**Table 1:** Descriptive Statistics by Gender, Age

		N (%)	Mean	SD
	All	348		
gender	Male	232 (66.7)		
	female	116 (33.3)		
age	17.00	7 (2.0)	20.37	1.33
	18.00	18 (5.2)		
	19.00	65 (18.7)		
	20.00	97 (27.9)		
	21.00	83 (23.9)		
	22.00	66 (19.0)		
	23.00	11 (3.2)		
	24.00	1 (0.3)		

### Questionnaires

#### IOTN

About young adults perceived Aesthetic Component (young adult’s AC), everyone was shown the 10 photographs of the IOTN and was asked to select the photograph that best represented his/her dental appearance. The Aesthetic Component assessed by orthodontists (orthodontist’s AC) of the IOTN and the Dental Health Component (DHC) was recorded by the author. AC ranks attractiveness of dental appearance on a scale of 1–10, from the least attractive to the most attractive. DHC ranks malocclusions on a scale of 1–5 according to the severity of occlusion traits. AC grades 1 to 4 and DHC grades 1 and 2 represent little or no need for treatment, AC grades 5 to 7 and DHC grade 3 a borderline need, and finally AC grades 8 to 10 and DHC grades 4 and 5 a definite need for treatment.

#### Personality traits

The Chinese version of EPQ [21, 22], an adaption of the personality traits, was used to assess Chinese respondent. Eighty eight items consisted of yes/no questions were shown to the participants. The whole scale includes four factors, which are extroversion/introversion (EPQ-E, representing sociability, liveliness, and positive), Neuroticism (EPQ-N, representing emotional instability and anxiousness), psychoticism (EPQ-P, representing tough-mindedness, aggressiveness, coldness, and egocentricity), and lie (EPQ-L). The EPQ-L is a control scale reflecting the individual level of social naivety [21, 23].

### Statistical analyses

Cohen’s kappa test was used to assess the consistency of the AC grade determined by young adults and orthodontists, as well as the agreement of orthodontist’s AC and DHC [24]. The chi-square test was applied to test distribution differences between the young adult’s AC and the orthodontist’s AC of the IOTN, and the differences between males and females. Spearman’s correlation coefficient was used to identify whether there is a correlation among young adult’s AC, orthodontist’s AC, DHC, personality traits and gender [25]. The statistical analysis was performed using the Statistical Package for Social Sciences (SPSS, version 19.0 for Windows; Chicago, IL, USA). A *p* value less than or equal to 0.05 was considered statistically significant (*p* ≤ 0.05).

## Results

A statistically significant level of agreement was observed between young adults and orthodontist perception (kappa = 0.14). A significant positive relationship existed between the young adult and orthodontist’s AC scores (*r* = 0.275, *p* < 0.001), and between young adult’s AC and DHC (*r* = 0.195, *p* < 0.001). The orthodontist’s AC was strongly related with the normative need (*r* = 0.743, *p* < 0.001), but only a moderate agreement was observed (kappa = 0.332, *p* < 0.001). Correlations between the young adult’s AC and gender (*r* = 0.143, *p* = 0.007), EPQ-E were also observed (*r* = − 0.112, *p* = 0.037).

The results of the frequency measured according to young adult’s AC, orthodontist’s AC and DHC of the IOTN are shown in Table 2. Orthodontists perceived 63.8% to be in mild category with regards to AC (grade 1 to grade 4), which was lower than young adult’s self-perception, 30.2% to be in moderate category (grade 5 to grade 7), 6% to be in sever category (grade 8 to grade 10), which is higher compared to young adult’s self-perception. According to the normative need DHC, 36.5% of the participants were borderline need to seek orthodontic treatment, 24.1% was in a definite need for treatment. Table 3 shows the distribution of the perceived treatment need by both young adults and the orthodontists. The kappa test (kappa = 0.14, *p* < 0.001) shows a certain level of agreement between young adults and orthodontists, even though the agreement is weak. A moderate agreement was observed between orthodontists perceived treatment need and normative need for treatment (kappa = 0.332, *p* < 0.001) (Table 4). DHC was more critical than perceived judgments according to orthodontist’s AC. There was a correlation between the components of IOTN, young adult’s AC, orthodontist’s AC and DHC (Table 5). A significant positive relationship (*p* < 0.001) between the young adult’s AC and orthodontist’s AC scores (*r* = 0.275); young adult’s AC and the normative need DHC (*r* = 0.195), the orthodontist perception and the normative need (*r* = 0.743). Table 6 shows the correlations between the young adult’s AC and gender (*r* = 0.143, *p* = 0.007) and, EPQ-E (*r* = − 0.112, *p* = 0.037).

**Table 2:** Frequency of treatment need according to AC and DHC

IOTN	DHC (n %)	Young adult’s AC (n %)	Orthodontist’s AC (n %)
1	137 (39.4)	313 (89.9)	222 (63.8)
2	127 (36.5)	23 (6.6)	105 (30.2)
3	84 (24.1)	12 (3.4)	21 (6.0)
Total	348	348	348

1 mean: little or no need for treatment; 2 mean: borderline need; 3 mean: definite need for treatment

**Table 3:** Distribution of young adults perceived treatment need in relation to orthodontists perceived treatment need as evaluated by AC

		Orthodontist’s AC			Total
		1.00	2.00	3.00	
Young adult’s AC	1.00	213	86	14	313
	2.00	4	13	6	23
	3.00	5	6	1	12
Total		222	105	21	348

1 mean: little or no need for treatment; 2 mean: borderline need; 3 mean: definite need for treatment; Kappa = 0.14, *p* < 0.001

**Table 4:** Distribution of orthodontist’s perceived treatment need in relation to normative treatment need

		Orthodontist’s AC			Total
		1.00	2.00	3.00	
DHC	1.00	136	1	0	137
	2.00	81	46	0	127
	3.00	5	58	21	84
Total		222	105	21	348

1 mean: little or no need for treatment; 2 mean: borderline need; 3 mean: definite need for treatment; Kappa = 0.332, *p* < 0.001

**Table 5:** Correlation between orthodontist and young adult’s perception and normative need

		DHC	Orthodontist’s AC
Spearman’s correlation	young adult’s AC	0.195**	0.275**
	Orthodontist’s AC	0.743**	1.000

** *p* < 0.001, * *p* < 0.05

**Table 6:** Factors that correlate patient perception by IONT-AC

		Gender	T score of E	T score of P	T score of N
Spearman’s correlation	Young adult’s	.143**	−.112*	0.027	0.064
		0.007	.037	0.621	0.235

** *p* < 0.001, *p < 0.05

## Discussion

The aim of this study was to assess the agreement between orthodontists and the young adult’s perception regarding to the IOTN-AC. We found that young adults tend to be less critical in assessing orthodontic treatment needs than orthodontists. However, the orthodontist’s AC reflecting subjective treatment need is strongly connected to the normative need. Due to the complex doctor-patient relationship, orthodontists in China must consider this consistency [26].

This study focused on the agreement between orthodontists and young adult’s perception of dental malocclusions. Although an agreement in perception was observed, the value indicated that it was not clinically relevant. Orthodontists perceived 36.2% of the young adults to be in need for treatment, but only 11% of the young adults were considered that need receive orthodontic treatment. The contrast exits because the present group presumably has less awareness of dental function as well as dental aesthetics. Many studies have found that the motivation of the most people seeking orthodontic treatment is related to the appearance improvement and the desire for attractiveness [27, 28]. In the present study, the findings indicate that the opinion of the young adults does not align with the orthodontists. The young adults are not capable of understanding the severity of their presenting conditions. Therefore, better communication between orthodontists and young adults is necessary. Before providing treatment plans, it’s better for orthodontists to explain to the young adults their dental conditions and why they have to take treatment into consideration. This communication helps to improve the patient’s trust in their doctor in-charge and to obtain the compliance of the patient in the long-term orthodontic treatment duration.

It had been investigated that the choice for an orthodontic treatment demand were influenced by the desire for improvement in the appearance [3]. In this study, we try to explore the factors such as gender and personality traits that influence the subjective perception of aesthetic component among Chinese young adults. It is suggested that Chinese females are more critical with their dental appearance than the males. The result that females have a higher perception of aesthetic component is also supported by some other studies [29]. The study also shows that EPQ-E has some extent influence on subjective perception of aesthetic components. Young adults with higher introversion emotion are more critical with their dental aesthetic.

This study indicates that orthodontists should pay more attention to the perception discrepancy of aesthetics when young adults seek orthodontic treatment. Orthodontists should consider individual differences such as gender and personality traits. Be aware of the personality traits associated with the patient’s mental state and help the orthodontists communicate with the patient during the treatment [30]. The psychological status of the patient before any orthodontic treatment should not be overlooked, since it can be closely related to certain difficulties during the treatment, including the patient’s discomfort and dissatisfaction [31, 32]. In the future, we will recruit samples from different cities to further explore and identify these findings in multi-center studies. In addition, the difference in dental aesthetic understanding between the patient and the orthodontist should be carefully considered in the patient’s doctor’s relationship. Also, we will try to investigate whether the psychological state will interfere with the outcome of orthodontic treatment.

## Conclusions

Young adults tend to be less critical in assessing orthodontic treatment needs than orthodontists, and the orthodontist’s AC reflecting subjective treatment need is strongly connected to the normative need. Furthermore, the adult’s perception of aesthetic component is affected by gender and personality traits.

## Abbreviations

AC: Aesthetic components
DHC: Dental Health Components
EPQ: Eysenck Personality Questionnaire
IOTN: the Index of Orthodontic Treatment Need
